# Effect of *Ocimum sanctum*, ascorbic acid, and verapamil on macrophage function and oxidative stress in mice exposed to cocaine

**DOI:** 10.4103/0253-7613.55210

**Published:** 2009-06

**Authors:** S.K. Bhattacharya, N. Rathi, P. Mahajan, A.K. Tripathi, K.R. Paudel, G.P. Rauniar, B.P. Das

**Affiliations:** Department of Pharmacology, B. P. Koirala Institute of Health Sciences, Dharan, Nepal, India; 1 Department of Pharmacology, University College of Medical Sciences, Delhi - 110 095, India; 2 Department of Biochemistry, University College of Medical Sciences, Delhi - 110 095, India

**Keywords:** Ascorbic acid, cocaine, macrophage, *Ocimum sanctum*, verapamil

## Abstract

**Objective:**

To investigate the effect of *Ocimum sanctum*, ascorbic acid, and verapamil on macrophage function and oxidative stress in experimental animals exposed to cocaine.

**Materials and Methods:**

Mice were used in this study and were divided randomly into different groups of six animals each. They were either treated with intraperitoneal injection of saline or cocaine hydrochloride or an oral feeding of oil of *Ocimum sanctum*, ascorbic acid or verapamil, or both (ascorbic acid and verapamil), and were evaluated for a respiratory burst of macrophages, superoxide and nitric oxide (NO) production, estimation of TNF-α in the serum and supernatant of cultured macrophages, estimation of lipid peroxidation (malondialdehyde- MDA) in the serum, and superoxide dismutase activity in the erythrocytes.

**Results:**

Unstimulated respiratory burst as well as superoxide production was enhanced on treatment with cocaine and all the three drugs were found to attenuate this enhancement. The bactericidal capacity of macrophages decreased significantly on chronic cocaine exposure, as it was associated with decreased respiratory burst and superoxide production. There was a significant decrease in NO production by macrophages on chronic cocaine exposure and all the test drugs were found to restore nitrite formation to a normal level. There was an increase in the malonylodialdehyde (MDA) level and decrease in the superoxide dismutase level on chronic cocaine exposure, and all the three drugs effectively decreased the MDA level and increased superoxide dismutase level. There was an increase in serum TNF-α on chronic cocaine exposure, which was decreased significantly by ascorbic acid and verapamil.

**Conclusion:**

*O. sanctum*, ascorbic acid, and verapamil were equally effective in improving the macrophage function and reducing oxidative stress. These findings suggested that *O. sanctum*, ascorbic acid, and verapamil attenuated acute and chronic cocaine-mediated effects.

## Introduction

Natives of certain parts of South America started the use of coca (Erythroxylon coca) leaf for euphoric purposes centuries ago. It was isolated by Albert Neimann (1860), who tested this compound and found that it caused numbing of the tongue. Sigmund Freud studied cocaine's physiological actions, and Carl Koller introduced it into clinical practice in 1884 as a topical anesthetic for ophthalmic surgeries. At that time, people discovered its euphoric properties and it began to be fairly largely used in the USA for this purpose. It was also known by local names, such as, Coke, Snow, Gold dust, and Lady. The successful use of cocaine for producing local anesthesia began to be increasingly appreciated in the early parts of the twentieth century, and it was considered worthwhile to produce it synthetically.[1] Today cocaine abuse has become a major health hazard worldwide. Cocaine and heroin are two of the most common parenterally abused drugs and cocaine users are seen to be particularly susceptible to opportunistic infections. Several workers have demonstrated the existing relationship between the use of drugs of abuse and the occurrence of infections.[2–4]

Macrophages are essential cells in a host defense. They synthesize and release a variety of active biomolecules within minutes to hours after stimulation. These molecules include reactive oxygen intermediates, reactive nitrogen intermediates,[5] cytolytic proteases, and a tumor necrosis factor,[6] and are involved in non-specific defense mechanisms including phagocytosis and killing of microorganisms and foreign bodies.

Exposure to cocaine has been shown to influence immunity at multiple points, which results in an abnormality in antibody production[7] and aberrant lymphocyte functioning.[8] Although, no clear picture emerges on the role of cocaine in the immune function yet, macrophage dysfunction is believed to be the major cause of immunosuppression in cocaine abusers.[9,10]

The Holy basil or Tulsi has been used as an expectorant, tonic, rejuvenator or a vitalizer, to induce longevity and a disease-free state. It has been proposed that it has an immunostimulant action, which might be due to its adaptogenic action.[11] Verapamil is a phenylalkylamine class of calcium channel blocker more selective for L type of calcium channels and it has been shown to possess an immunomodulatory effect at the cellular level. By blocking calcium entry into the cells, verapamil has been known to regulate the transcription of a large number of pro-inflammatory and anti-inflammatory cytokines.[12] There have been numerous reports during the last several years regarding the effects of ascorbic acid on macrophages. Mononuclear phagocytes contain high concentrations of ascorbate (2.0 μg/mg protein) with both peritoneal and alveolar macrophages, being rich in ascorbate.[13]

The assessment of the immunomodulatory role of *O. sanctum*, ascorbic acid, and verapamil could provide a basis for future attempts to improve the *in vivo* immune system by administration of these antioxidants[12,13] in oxidative stress situations, such as, the use of cocaine in addicts. So, the aim of the present study is to investigate the effect of *O. sanctum,* ascorbic acid, and verapamil on the macrophage function and oxidative stress in mice exposed to cocaine.

## Materials and Methods

### Drugs and chemicals

Cocaine hydrochloride was supplied by M/S Sigma Chemical Company, USA, after obtaining permission from the Narcotic Commissioner of India. A solution of drug in saline was administered intraperitoneally (i.p.) at a dose of 10 mg/kg/day.

The dried seeds of *O. sanctum* were collected from Maidan Garhi, New Delhi, and were crushed and cold macerated in petroleum ether (40 – 60°C) for three days. Petroleum ether was evaporated from the extract and the oil was filtered. Swiss albino mice were subjected to oral feeding of this oil.

Ascorbic acid was purchased as CELIN-500 mg tablets. They were ground using a pestle and mortar, suspended in 0.9% saline, and administered at a dose of 200 mg/kg/day.

Verapamil was procured as Calaptin-40 mg tablets. It was ground in a pestle and mortar, suspended in 0.9% saline and administered at a dose of 20 mg/kg/day.

### Animals

Male Swiss albino mice (20-30 g) were used for the study. The animals were taken from the Central Animal Facilities of UCMS and GTBH and the All India Institute of Medical Sciences, Delhi, after obtaining permission from the Institutional Ethics Committee for the care of animals. The animals were randomly divided into groups of six animals each and were kept under standard laboratory conditions in plastic cages and were provided food and water *ad libitum*. The mice were either treated with intraperitoneal injection of saline or cocaine or an oral feeding of oil of *O. sanctum*, ascorbic acid, or verapamil, or both.

### Method

Two models of cocaine-treated mice were studied.

### Acute model

Mice were treated either with saline or cocaine plus test drugs for one day and the effect was studied after four hours. The mice were further divided into five groups with six mice each as follows: group 1 - saline; group 2 - cocaine hydrochloride (10 mg/kg i.p.), groups 3 to 5 - cocaine and test drugs. The mice received one dose of the test drug (oral); either oil of *O. sanctum* (3 ml/kg) or verapamil (20 mg/kg) or ascorbic acid (200 mg/kg) plus cocaine hydrochloride (10 mg/kg i.p.), one hour after the test drug.

### Chronic model

The mice were treated with saline or cocaine or cocaine plus test drugs for seven days and the test parameters were evaluated after 14 hours of the last dose. In this model, the mice were further divided into five groups with six mice in each, as follows: group 1 - saline (i.p.) once daily; group 2 - cocaine hydrochloride (10 mg/kg i.p.) once daily; groups 3 to 5 - cocaine plus test drugs (oral); either oil of *O. sanctum* (3 ml/kg) or verapamil (20 mg/kg) or ascorbic acid (200 mg/kg) plus cocaine hydrochloride (10 mg/kg i.p.), one hour after the test drug.

### Blood sample collection

Blood samples were collected from the retro-orbital plexus of the veins of mice held in the left hand, such that the index finger and thumb held the skin of the back of the neck and the middle finger stabilized the jaw. A capillary tube was taken and pushed through the medial canthus of the bulged eyeball, in a medial and backward direction. The tube reached the retro-orbital place puncturing the retro-orbital plexus. The blood that flowed into the tube was then collected in two tubes, one of which contained an appropriate amount of heparin.

### Preparation of serum

After collection of blood, it was allowed to settle in the test tube. Two to three hours later, the sample that was already somewhat separated into serum, was centrifuged at 3000 rpm for 5 minutes. The serum thus obtained was then pipetted out into a small test tube and was used for the estimation of serum lipids and serum MDA levels.

### Preparation of platelet rich plasma and hemosylate

After collection of the whole blood (heparinized), it was centrifuged at 2500 rmp for 10 minutes. The RBCs settled down. The supernatant was the platelet rich plasma (PRP) and was used for the estimation of serum lipid peroxides. For preparing hemosylate, 0.5 ml of RBC was taken and washed with 3 ml of normal saline, thrice. At the end of the third wash, the saline was removed and 1.5 ml of cold water was utilized for the estimation of superoxide dismutase activity (SODA).

### Isolation of macrophages

Blood was collected from the treated mice and they were anesthetized using chloroform. The mice were then subjected to peritoneal lavage using 5 ml of ice-cold Hank's balanced salt solution (HBSS). The animals were gently shaken so that proper mixing of the peritoneal exudates could take place. The cavity was then opened using a midline incision and lavage fluid collected in a petri dish. Peritoneal lavage was again repeated using 2 ml of HBSS and lavage fluid from all the animals was pooled in the same petri dish. The fluid was then incubated at 37°C under 5% CO_2_ for 30 minutes. After 30 minutes the nonadherent cells and debris were washed away by vigorous shaking and the supernatant drained off. This procedure was repeated twice. The adherent cells were then collected in an ice-cold solution of HBSS using a rubber policeman. The cells were counted and a regular suspension was made such that it contained 2.5 × 10^6^ cells/ml. The cells were tested for their viability with the help of the tryptan blue 0.5% exclusion test. Only suspensions showing > 90% viable cells were taken for the experiments.

The following parameters were evaluated in the study by using serum, hemosylate, or macrophages; respiratory burst of macrophages, superoxide production in macrophages, nitric oxide production in macrophages, lipid peroxidation in blood, SODA in blood and tissue necrosis factor-alpha (TNF-α) in serum, and the supernatant of cultured macrophages.

### Assay of respiratory burst by nitroblue tetrazolium (NBT) reduction

The assay represents a modification of a quantitative NBT reduction assay, for use in microplates. The amount of reduced NBT is measured directly in the cells present in the wells, with the aid of an ELISA reader, at a 550 nm filter.[14]

### Assay of superoxide anion (O_2_^-^)

The assay is based on the reduction of ferricytochrome C by superoxide ions (O_2_^-^). The specificity of the reduction is controlled by its inhibition by superoxide dismutase (SOD).[14]

### Estimation of nitric oxide (NO)

Estimation of nitric oxide produced by macrophages was done by following the method of Ding *et al*. (1988).[15]

### Estimation of lipid peroxides in serum

Serum MDA level is a quantitative measure of serum lipid peroxides. The thiobarbituric acid (TBA) method, which is a colorimetric method, described by Slater and Sawyer and later modified by Satosh (1978)[16] was used for the quantitative analysis of serum lipids.

### Estimation of erythrocytes superoxide dismutase activity (SODA)

Erythrocytic SODA was determined by the pyrogallo auto-oxidation method described by Marklund and later modified by Nandi and Chatterjee (1988).[17]

### Estimation of TNF-α

TNF-α was estimated using the Enzyme-Linked-Immuno-Sorbent Assay (ELISA) kit supplied by Diaclone Research, France. The murine TNF-α kit (mTNF-α) was a solid phase sandwich ELISA. A monoclonal antibody specific for mTNF-α had been coated onto the wells of the microtiter strips. The antigen and a biotinylated polyclonal antibody specific for mTNF-α were simultaneously incubated. The revelation step included Streptavidin-Horse Radish peroxidase and tetramethylbenzidine (TMB) as chromogen.

### Statistical analysis

Analysis of data was done using one-way ANOVA followed by Tukey's test. *P* value < 0.05 was considered significant.

## Results

### Respiratory burst

It was assayed by following the method involving NBT reduction, using either resting macrophages (Mφ) or in the presence of a stimulant Phorbol Myristate Acetate (PMA) and the results were expressed as OD_540_/10^6^ cells/30 minutes. On treatment with a single dose of cocaine, there was a significant increase in respiratory burst, in the absence of a stimulant. When treated with cocaine along with *O. sanctum,* ascorbic acid, or verapamil, an unstimulated respiratory burst reduced the control values and was significant when compared to the cocaine-treated group. On stimulation with PMA, a respiratory burst in cocaine-treated macrophages did not show any change with respect to control, but co-treatment of cocaine with drugs reduced the respiratory burst of macrophages significantly. In chronic cocaine treatment, the unstimulated respiratory burst decreased significantly when compared to the cocaine-treated group with *O. sanctum,* ascorbic acid, or verapamil. The respiratory burst of PMA-stimulated macrophages on the other hand was found to be significantly decreased on chronic cocaine treatment and it was restored to normalcy, significantly, when treated with *O. sanctum*, [Table 1].

**Table 1:** Respiratory burst activity in various groups on acute and chronic treatment with cocaine ± drugs

*Acute model*	*Chronic model*			
	
*Groups*	*Respiratory burst (OD_540_/10^6^ cells/ 30 min)*		*Respiratory burst (OD_540_/10^6^ cells/ 30 min)*	
		
	*Unstimulated Mφ*	*PMA Stimulated Mφ*	*Unstimulated Mφ*	*PMA Stimulated Mφ*
Control	0.07 ± 0.02	0.45 ± 0.05	0.07 ± 0.02	0.57 ± 0.06
Cocaine	0.12 ± 0.02^a^	0.44 ± 0.06	0.20 ± 0.03^d^	0.28 ± 0.11^f^
Cocaine + *O. sanctum*	0.08 ± 0.01^b^	0.30 ± 0.03^c^	0.09 ± 0.04^e^	0.49 ± 0.14^g^
Cocaine + ascorbic acid	0.07 ± 0.02^b^	0.26 ± 0.03^c^	0.11 ± 0.02^e^	0.31 ± 0.04
Cocaine + verapamil	0.08 ± 0.01^b^	0.28 ± 0.08^c^	0.07 ± 0.02^e^	0.39 ± 0.05

Values are Mean ± SEM. n = 6 in each group.  *P* values; acute model- a < 0.001 vs. control, b < 0.01 cocaine In unstimulated respiratory burst, c < 0.001 vs. control In PMA stimulated respiratory burst and chronic model - d < 0.001 vs. control, e < 0.001 vs. cocaine in unstimulated respiratory burst, f < 0.001 vs. control, g < 0.001 vs. cocaine in PMA stimulated respiratory burst. One-way ANOVA followed by Tukey's test. Mφ- macrophage. PMA- Phorbol Myristate Acetate

### Superoxide production

The assay is based on reduction of ferricytochrome C by superoxide ions (O_2_^-^). In an acute model, unstimulated superoxide production was reduced significantly by ascorbic acid when compared to cocaine, whereas, cocaine had no effect in PMA-stimulated superoxide release. In a chronic model, treatment with *O. sanctum,* ascorbic acid, or verapamil decreased significantly both in the unstimulated and PMA-stimulated macrophage superoxide production when compared to cocaine, [Table 2].

**Table 2:** Superoxide production in various groups on acute and chronic treatment with cocaine ± drugs

*Acute model*	*Chronic model*			
	
*Groups*	Superoxide production *(nM/10^6^ cells/ 30 min)*		Superoxide production *(nM/10^6^ cells/ 30 min)*	
		
	*Unstimulated Mφ*	*PMA Stimulated Mφ*	*Unstimulated Mφ*	*PMA Stimulated Mφ*
Control	0.76 ± 0.23	3.39 ± 0.96	0.64 ± 0.07	4.47 ± 0.64
Cocaine	1.62 ± 0.32^a^	4.21 ± 0.59	1.77 ± 0.55	1.80 ± 0.50^d^
Cocaine + *O. sanctum*	1.03 ± 0.43	3.85 ± 0.62	0.80 ± 0.12^c^	4.12 ± 0.57^e^
Cocaine + ascorbic acid	0.88 ± 0.24^b^	3.96 ± 0.40	0.92 ± 0.34^c^	3.74 ± 0.41^e^
Cocaine + verapamil	1.09 ± 0.32	3.81 ± 0.89	0.94 ± 0.16^c^	4.00 ± 0.44^e^

Values are Mean ± SEM. n = 6 in each group.  *P* values; acute model- a < 0.001 vs. control, b < 0.01 cocaine In unstimulated Superoxide production, and chronic model- c < 0.001 vs. cocaine in Unstimulated Superoxide production, d < 0.001 vs. control, e < 0.001 vs. cocaine in PMA stimulated Superoxide production. One-way ANOVA followed by Tukey's test. Mφ- macrophage. PMA- Phorbol Myristate Acetate

### Nitric oxide (NO) production

Nitric oxide produced by macrophages in the culture media was estimated by measuring the nitrite content of the sample using the Griess reagent. In the acute model, there was no significant effect in NO production, whereas, in the chronic model cocaine significantly reduced NO production and was significantly restored to normal values either with *O. sanctum* or ascorbic acid or verapamil, [Table 3].

**Table 3:** Nitric oxide (NO) production by cultured macrophages in various groups on acute and chronic treatment with cocaine ± drugs

*Acute model*	*Chronic model*	
	
*Groups*	*Nitrite secretion (μM/10^6^)*	*Nitrite secretion (μM/10^6^)*
Control	3.11 ± 0.82	3.57 ± 0.76
Cocaine	2.81 ± 0.85	1.97 ± 0.72^a^
Cocaine + *O.sanctum*	3.78 ± 1.43	4.06 ± 0.95^b^
Cocaine + ascorbic acid	4.11 ± 1.25	3.31 ± 0.92^b^
Cocaine + verapamil	4.42 ± 1.06	3.80 ± 0.86^b^

Values are Mean ± SEM. n = 6 in each group. *P* values; a < 0.001 vs. control, b < 0.001 vs. cocaine. One-way ANOVA followed by Tukey's test.

### TNF-α level

It was estimated using the sandwich ELISA technique. Serum and supernatant from cultured macrophages were estimated for their TNF-α content. Both in the acute and chronic models, cocaine significantly increased the TNF-α secretion by macrophages and it was reduced significantly toward the normalcy with either ascorbic acid or verapamil treatment, [Table 4].

**Table 4:** Estimation of TNF-α level in various arouos on acute and chronic treatment with cocaine ± druas

*Acute model*	*Chronic model*			
	
*Groups*	*TNF- α level (pg/ml)*		*TNF- α level (pg/ml)*	
		
	*Supernatant*	*Serum*	*Supernatant*	*Serum*
Control	35.40 ± 3.35	24.62 ± 2.33	35.29 ± 2.82	25.32 ± 2.09
Cocaine	44.73 ± 2.24^a^	27.88 ± 2.66	50.56 ± 3.69^c^	32.15 ± 2.81
Cocaine + *O.sanctum*	40.35 ± 2.54	25.42 ± 1.88	26.24 ± 1.73	21.25 ± 2.49
Cocaine + ascorbic acid	35.45 ± 2.46^b^	28.57 ± 2.07	29.31 ± 1.38^d^	19.90 ± 2.64
Cocaine + verapamil	35.58 ± 2.22^b^	27.83 ± 2.08	29.93 ± 3.20^d^	17.34 ± 1.90

Values are Mean ± SEM. n = 6 in each group.  *P* values; acute model- a < 0.001 vs. control, b < 0.01 vs. cocaine, and chronic model- c < 0.001 vs. control, d < 0.001 vs. cocaine. One-way ANOVA followed by Tukey's test.

### Oxidative stress

The measurement of serum MDA levels and erythrocyte SODA was used to assess the oxidative status of the animals. Erythrocytic superoxide dismutase was assessed by quantifying the amount of erythrocytic extract required to bring about an inhibition of auto-oxidation of pyrogallol. In an acute model, treatment either with ascorbic acid or verapamil reduced cocaine-induced MDA levels and was significant. However, there was no significant effect on the superoxide dismutase activity with any of the three test drugs. In a chronic model, all the three test drugs reduced both the cocaine-induced MDA levels and superoxide dismutase activity significantly, [Table 5].

**Table 5:** Oxidative status of various groups on acute and chronic treatment with cocaine ± drugs

*Acute model*	*Chronic model*			
	
*Groups*	*MDA levels (nM/ml)*	*Superoxide dismutase activity (U/gmHb)*	*MDA levels (nM/ml)*	*Superoxide dismutase activity (U/gmHb)*
Control	3.43 ± 0.46	1414 ± 118	3.47 ± 0.60	1480 ± 131
Cocaine	4.01 ± 0.52	1471 ± 148	6.70 ± 0.78^b^	1163 ± 125^d^
Cocaine + *O.sanctum*	4.09 ± 0.30	1221 ± 141	4.11 ± 0.59^c^	2249 ± 323^e^
Cocaine + ascorbic acid	3.57 ± 0.56^a^	1208 ± 122	3.69 ± 0.54^c^	1589 ± 209^e^
Cocaine+verapamil	3.40 ± 0.59^a^	1297 ± 102	3.48 ± 0.31^c^	1748 ± 279^e^

Values are Mean ± SEM. n = 6 in each group.  *P* values; acute model- a < 0.001 vs. cocaine, and chronic model- b < 0.01 vs. control, c < 0.001 vs. cocaine in MDA levels, d < 0.001 vs. control, e < 0.001 vs. control in superoxide dismutase activity. One-way ANOVA followed by Tukey's test. MDA- serum malondialdehyde.

## Discussion

In the present study, the effect of acute and chronic exposure of cocaine on macrophage function and oxidative test parameters was investigated. Further, we explored the probability of whether some known antioxidant drugs (ascorbic acid and verapamil)[12,13] and natural plant products (*O. sanctum*) could attenuate the effect of acute and chronic exposure of cocaine with respect to its production of reactive oxygen and reactive nitrogen species and oxidative stress.

Cocaine enhanced the respiratory burst of macrophages, both in acute and chronic exposure, and it indicated an activated state of macrophages. Similarly, it increased the amount of superoxide release without stimulation, which might be one of the causes of cocaine-related multiorgan toxicity.[9,18] On the other hand superoxide release from the macrophage in response to pathogen / mitogenic stimulus was suppressed on chronic exposure. This is believed to be responsible for the poor bactericidal activity of macrophages, leading to infectious episodes in cocaine addicts.[2–4] NO is an important signaling and effector molecule that mediates a number of physiological and pathological processes. In the present study, acute exposure of cocaine to macrophages did not have any significant NO lowering effect and was similar to the previous findings,[10] whereas, chronic exposure decreased NO production significantly.

Although similar studies on the effect of chronic cocaine exposure to macrophages are unavailable, a recent study[19] has shown that TGF-β, a cytokine that inhibits various cell functions, is induced when exposed to cocaine. It is possible that TGF-β induction by cocaine may interfere with superoxide and NO production by macrophages.

Supernatant TNF-α increased significantly in both acute and chronic cocaine exposures. The increase in the unstimulated respiratory burst and superoxide production on exposure to chronic doses of cocaine may be due to increased TNF-α secretion, and the latter is mediated at the transcription level by cocaine and its metabolites.[20] Oxidative stress parameters, which were evaluated, included serum MDA levels, indicative of lipid peroxidation and erythrocytic superoxide dismutase representing a superoxide scavenging system. Results of the study showed a significant increase in the serum MDA levels on chronic cocaine exposure, which was in consonance with a past study,[21] and the significantly low level of erythrocytic superoxide dismutase activity observed in this study is possibly associated with a general low antioxidant status, due to chronic cocaine exposure.

Results obtained in this study showed that administration of the oil of *O. sanctum* arrested the cocaine-mediated increase in respiratory burst, PMA-stimulated superoxide production, and serum MDA levels, and it also restored the SODA decreased by chronic cocaine exposure. Although the exact mechanism of such effects of *O. sanctum* was not clear, induction of both enzymatic and non-enzymatic antioxidant mechanisms were reported as an explanation for the antioxidant properties shown by *O. sanctum*.[22]

Coadministration of ascorbic acid significantly reduced the cocaine-induced respiratory burst, unstimulated superoxide production, TNF-α secretion, and serum MDA levels, whereas, it significantly increased the SODA and NO secretion in chronic cocaine exposure. The mechanism of the increased level of superoxide dismutase (SOD) by ascorbic acid was not clearly known, whereas, the increased NO secretion might possibly be mediated through the induction of an inducible NO synthase (iNOS) gene.[23]

Verapamil caused reduction of the unstimulated respiratory burst, superoxide production, TNF-α secretion, and oxidative stress induced by cocaine exposure. TNF-α is an important cytokine employed in the regulation of reactive oxygen species generation. Decrease in TNF-α production by verapamil was done by blocking the Ca^++^-dependent pathway, which was important for the synthesis and release of this cytokine.[12] Moreover, the antioxidant property of verapamil was because of the protection against membrane damage caused by a reactive species on account of a calcium independent pathway.[24]

## Conclusion

Cocaine increased respiratory burst of macrophages and superoxide release from macrophages, both in acute and chronic exposures, without stimulation. On the contrary, the respiratory burst of macrophages and superoxide release from the macrophage in response to pathogen / mitogenic stimulus was suppressed on chronic exposure. Therefore, exposure to cocaine caused functional inability of the macrophages to respond to pathogenic / mitogenic stimuli. Moreover, an increase in serum lipid peroxides and a decrease in the antioxidant enzyme superoxide dismutase, as also decreased NO production and increased TNF-α production have been observed with cocaine exposure. Therefore, such effects of cocaine are responsible for immunosuppression and possible organ toxicity in cocaine users. *O. sanctum, ascorbic acid,* and *verapamil* are effective in reversing cocaine-mediated macrophage dysfunctions and oxidative stress, thereby, reducing both the acute and chronic effects of cocaine.
